# Effect of Y on Recrystallization Behavior in Non-Oriented 4.5 wt% Si Steel Sheets

**DOI:** 10.3390/ma15124227

**Published:** 2022-06-15

**Authors:** Jing Qin, Haibin Zhao, Dongsheng Wang, Songlin Wang, Youwen Yang

**Affiliations:** 1 College of Mechanical Engineering, Tongling University, Tongling 244000, China; wsl-hf@126.com; 2 Faculty of Materials Metallurgy and Chemistry, Jiangxi University of Science and Technology, Ganzhou 341000, China; zhaohaibin1030@163.com; 3 School of Mechanical and Electrical Engineering, Jiangxi University of Science and Technology, Ganzhou 341000, China; yangyouwen@jxust.edu.cn

**Keywords:** 4.5 wt% Si steel, yttrium, recrystallization textures, segregation, inclusion

## Abstract

4.5 wt% Si steel sheets with four different yttrium (Y) contents (0, 0.006, 0.012 and 0.016 wt%) were fabricated by hot rolling, normalizing, warm rolling and a final annealing process. Y addition greatly weakened the γ -fiber (〈111〉//ND) texture and enhanced the {001} 〈130〉 and {114} 〈481〉 texture components, and the magnetic properties were improved related to the effects of Y on the recrystallized grain nucleation. Y segregation at the grain boundaries inhibited the nucleation of {111} oriented grains at grain boundaries, which was beneficial to the nucleation and growth of other oriented grains elsewhere. At the same rolling reduction, Y_2_O_2_S inclusion caused more stress concentration than Al_2_O_3_ inclusion. Y_2_O_2_S in deformed grains with low energy storage provided more preferential nucleation sites for {001} 〈130〉 and {114} 〈481〉 grains. Strong {001} 〈130〉 and {114} 〈481〉 recrystallization textures due to the high mobility were obtained in samples containing 0.012 wt% Y.

## 1. Introduction

Steel sheets with 4.5 wt% Si have excellent magnetic properties and good ductility compared with 6.5 wt% silicon steel, and they are suitable for manufacturing iron cores of motors at high frequency [1,2]. The motor operation urges the magnetic properties of electrical steels to be more stringent, and thus the steel purification, grain-size control and texture optimization for magnetic property improvement become more important. As is well known, the amount and size of inclusions directly affect the grain size and magnetic properties of non-oriented electrical steels [3]. 

It is demonstrated in a number of studies that rare earth (RE) can control the amount and distribution of inclusions by deoxygenation and desulfurization and can influence the grain size as well as optimize the recrystallization textures and finally improve the magnetic properties of silicon steel [4,5]. It has been reported that the inclusions density in the size range of 0–1 μm of the Fe-1.15%Si electrical steels with 0.011 wt% Ce was much lower than that of samples without Ce, and the average grain size of samples containing 0.011 wt% Ce was the largest. Therefore, Ce plays the role of reducing the number of fine inclusions and coarsening the size of inclusions [4]. 

The magnetic induction of silicon steel increased after adding 0.0066 wt% La, which was attributed to the enhancement of {001} 〈110〉 and {110} 〈110〉 textures [6]. La addition changed the micro-orientation relationship between the recrystallized grain and the matrix, and the nucleation sites of {001}〈100〉 (Cube) grains existed at the {112}〈132〉 and {110}〈110〉 matrix [7]. 

The intensity of the η-fiber (〈100〉//RD) texture increased, and the magnetic properties improved after adding 0.012 wt% Y in Fe-6.5%Si electrical steels as Y provided more nucleation sites for η-fiber texture by promoting the occurrence of shear bands [8]. Moreover, the tendency of RE segregation at the grain boundaries also affects the recrystallized grain nucleation. The moderate additions of La and Ce enhanced the favorable {113} 〈361〉 and λ -fiber (〈100〉//ND) textures and reduced the γ -fiber texture due to their segregation at grain boundaries inhibiting the nucleation of γ grains [9].

Much work thus far has focused on the effects of RE on the recrystallization behavior of silicon steel. However, the mechanism of texture optimization with RE addition remained unclear, and the effect of RE modification on recrystallization texture was rarely studied. In the present work, the recrystallization behavior of 4.5 wt% Si steels are studied by adding different Y content, and the influence mechanism of Y segregation and inclusion modification on recrystallization texture of 4.5 wt% Si steel is analyzed.

## 2. Experimental

To improve the magnetic properties of 4.5 wt% Si electrical steels, an appropriate amount of solid-state 99.99 wt% pure Y was added after refining to ensure the yield of rare earth by protective casting. Four kinds of 4.5 wt% Si steel ingots with 0, 0.006, 0.012 and 0.016 wt% Y were produced by a vacuum induction melting furnace, and the chemical composition was detected by inductively coupled plasma-atomic emission spectrometry (ICP-AES) as shown in Table 1. 

The ingots were forged into 21 mm thick and 65 mm wide slabs at 1050–900 °C, hot-rolled at 1100–800 °C to 2 mm-thick bands (80 mm width and 280 mm length), and then normalized at 950 °C and pickled. Afterwards, the normalized bands were cut and warm-rolled at 200 °C to 0.3 mm-thick sheets (80 mm width and 620 mm length). Finally, the sheets were annealed at 900 °C for 2 min under a mixed H_2_ and N_2_ atmosphere. The final sheets were tailored to rectangular samples (30 mm width and 300 mm length) for magnetic testing. The short-time annealing was to obtain fine grains to improve the strength of silicon steel sheets, which meets the requirements of high-frequency drive motor on mechanical properties. In order to study the stress concentration around different inclusions, the normalized sheets without Y and containing 0.012 wt% Y were cold-rolled to the same thickness of 1.6 mm.

The textures and local stress concentration around inclusions were measured by an Oxford Instruments HKL-Channel 5 electron backscattered diffraction (EBSD). The inclusions were analyzed by Zeiss ΣIGMA scanning electron microscope (SEM) and energy-dispersive spectroscopy (EDS). The transgranular fractures obtained at room temperature and intergranular fractures obtained at liquid nitrogen temperature were analyzed by X-ray photoelectron spectroscopy (XPS). The magnetic induction *B*_50_ (at 5000 A/m), and the core loss *P*_10/400_ (at a magnetic flux density of 1.0 T and 400 Hz) of samples were measured by an electrical steel tester (MPG200D).

## 3. Results and Discussion

### 3.1. Effect of Y Content on the Magnetic Properties and Textures of Final Sheets

**Figure 1:**
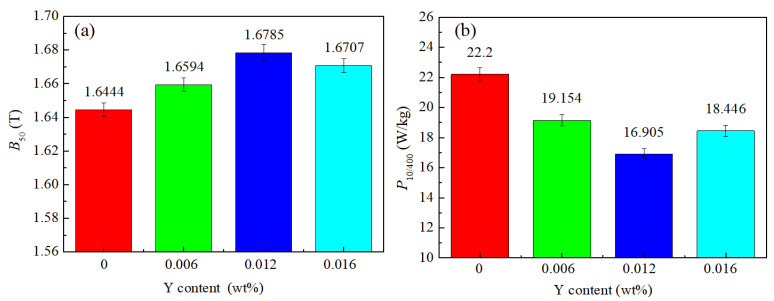
Magnetic properties of final sheets with different Y contents: (**a**) *B*_50_ and (**b**) *P*_10/400_.

Figure 1 shows the magnetic properties of the final sheets with different Y contents. It can be seen that, with the increase of Y content, the magnetic induction increased, and the core loss decreased to a certain extent. When the Y content was 0.012 wt%, the magnetic induction *B*_50_ reached the highest value (1.6785 T), and the core loss *P*_10/400_ reached the lowest value (16.905 W/kg). The magnetic properties of the 0.012 Y sample had the best magnetic properties among these samples.

**Figure 2:**
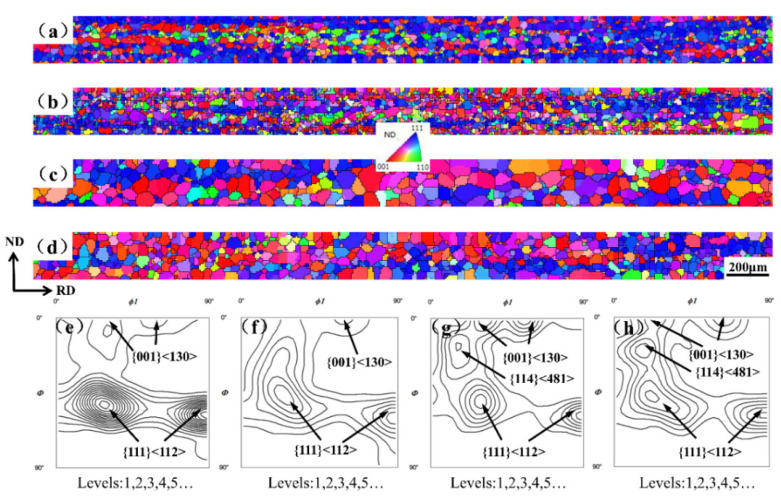
IPF maps, ODF at $\varphi_{2}$ = 45° section of final sheets with different Y: (**a**,**e**) 0 Y; (**b**,**f**) 0.006 Y; (**c**,**g**) 0.012 Y; and (**d**,**h**) 0.016 Y.

The IPF maps and ODF maps of the final sheets are shown in Figure 2. It can be seen that all the samples have been completely recrystallized. The average grain sizes of four samples with different Y contents were 34, 39, 57 and 50 μm, respectively. Adding an appropriate amount of Y can promote the grain growth in 4.5 wt% Si steel sheets; however, the grain growth was inhibited once the Y content was 0.016 wt%. The recrystallization texture of the 0Y sample was mainly composed of a strong {111} 〈112〉 texture and a weak {001} 〈130〉 texture. 

With adding 0.006 wt% Y, the {111} 〈112〉 texture was greatly weakened and the α* ({h,1,1} 〈1/h,1,2〉) texture closing to λ texture was enhanced to a degree. With adding 0.012 wt% Y, the γ texture intensity continued to weaken, and the {001} 〈130〉 texture and {114} 〈481〉 texture components increased significantly. When the content of Y reached 0.016 wt%, the γ texture components increased to a certain extent, and the {001} 〈130〉 texture was weakened; however, the {114}〈481〉 texture was further enhanced. 

### 3.2. Effect of Y Content on Texture Evolution

**Figure 3:**
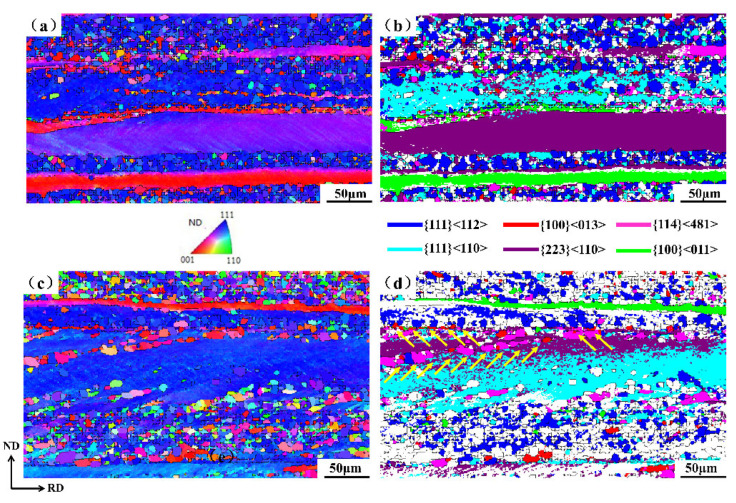
The IPF maps and texture component figures: (**a**,**b**) 0 Y and (**c**,**d**) 0.012 wt% Y.

In order to investigate the role of Y on recrystallization texture development, the origin of the nucleation of {111} grains and other competitive oriented grains in a partially recrystallized specimen was analyzed, and the textures were compared between 0 Y and 0.012 Y samples in Figure 3. As shown in Figure 3a,b, a large number of {111} 〈112〉 recrystallized grains nucleated at the grain boundaries and interior of the {111} 〈110〉 deformed grains, while the amount of {001} 〈130〉 and {114} 〈481〉 recrystallized grains was small, and the nucleation sites were scattered. 

However, in the 0.012 Y sample as shown in Figure 3c,d, {111} 〈112〉 grains were no longer the main recrystallized grains. Clearly, the number of the {100} 〈130〉 and {114} 〈481〉 recrystallized grains increased, and their nucleation sites were more diverse, nucleating at grain boundaries and within shear bands of {223} 〈110〉 deformed grains (indicated by yellow arrows in Figure 3d). Generally, γ grains nucleate at {111} deformed grains and at the grain boundaries between {111} and {hkl} 〈110〉 deformed grains [10,11]. 

The {111} recrystallized grains with nucleation advantage at the early recrystallization stage can consume α (〈110〉//RD) and λ deformed grains with low energy storage after consuming {111} deformed grains. As shown in Figure 3b, a large number of {111} recrystallized grains nucleated at the grain boundaries between {111} and {100} 〈110〉 deformed grains, and even some {111} recrystallized grains penetrated into the {100} matrix. In the 0.012Y sample, there were only a few other oriented grains nucleating at the boundary between {111} and {100} 〈110〉 deformed grains, which was related to the segregation of Y at grain boundaries. 

**Figure 4:**
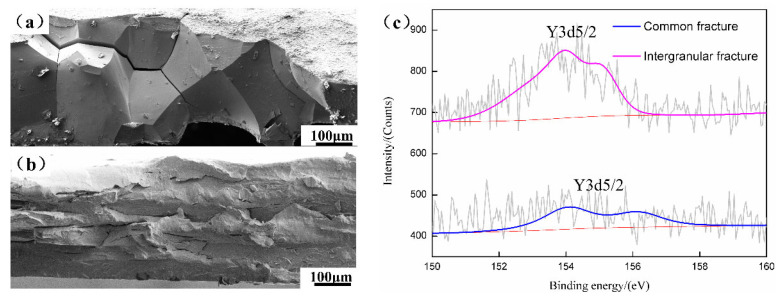
XPS in fracture of the final sheets containing 0.012 wt% Y: (**a**) intergranular fracture; (**b**) transgranular fracture; and (**c**) XPS map.

Due to the fact that the atomic diameter of Y (1.81 Å) is much larger than that of Fe (1.24 Å), and the solid solubility of Y in steel is low, it is more likely to segregate at grain boundaries with defects [8,12]. Figure 4a,b presents the intergranular fracture under impact at liquid nitrogen temperature and the transgranular fracture at room temperature of the final sheets containing 0.012 wt% Y. The XPS result of Y is shown in Figure 4c. According to the peak area, the Y content in the intergranular fracture was higher than that in the transgranular fracture. By comparison, it can be speculated that the Y atoms were enriched at the grain boundaries. Therefore, we considered that Y segregation at the grain boundaries restrained the preferential nucleation of γ grains and then reduced the γ texture. 

### 3.3. Effect of Y Inclusion on Recrystallization Behavior

An appropriate amount of Y can reduce the number of fine inclusions and increase the number of large-size inclusions, which significantly changes the grain size of the final sheets; however, effects on texture development were rarely found [8]. The static recrystallization process will be affected by the size, morphology and dispersion degree of the second phase particles in the matrix. We considered that large non-deformable particles promote recrystallization by particle-stimulated nucleation (PSN) and then affect the texture evolution [13]. 

**Figure 5:**
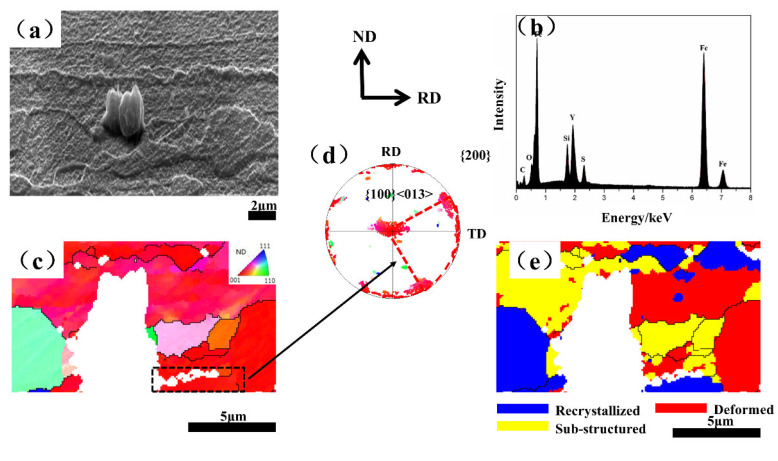
Recrystallization around a Y_2_O_2_S inclusion. (**a**) SEM micrograph; (**b**) EDS result; (**c**) IPF map; (**d**) {200} pole figure; and (**e**) recrystallization map.

Therefore, it is necessary to further study the effects of Y inclusion and modification on the texture development. Figure 5a,b shows the micrograph and EDS results around the inclusion in 2 μm in the 0.012 Y sample. Figure 5c–e shows the IPF map, the {200} pole figure and the recrystallization map, respectively. We found that the inclusion was Y_2_O_2_S, and the recrystallized grains mainly nucleated at the grain boundary and around the inclusion. The new grains at the grain boundary and inside the {100} deformed matrix were {001} 〈130〉 grains (indicated by a black frame in Figure 5c). 

**Figure 6:**
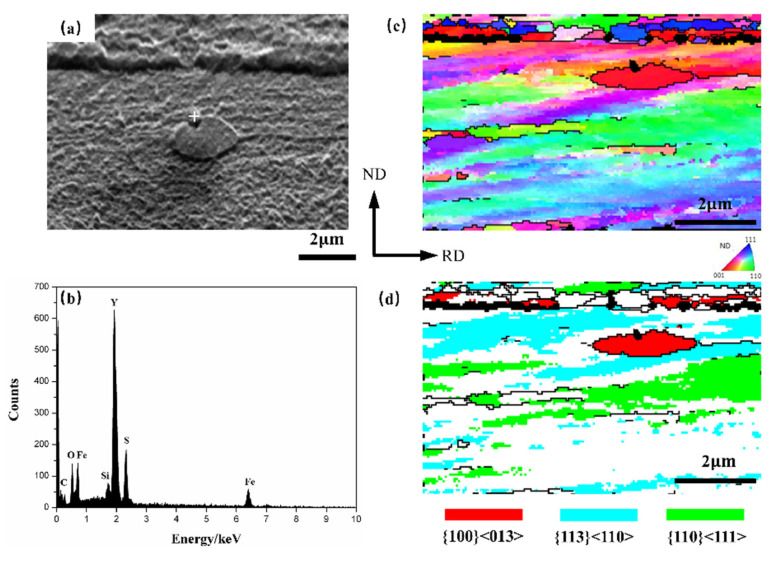
Recrystallization around a Y_2_O_2_S inclusion. (**a**) SEM micrograph; (**b**) EDS result; (**c**) IPF map; and (**d**) main texture components.

Similarly, as shown in Figure 6, {001} 〈130〉 grain nucleation was found in the interior of {113} 〈110〉 deformed matrix near Y_2_O_2_S inclusion at the early recrystallization stage in the 0.012 Y sample. It is generally believed that the energy storage of different deformed grains decreases with the sequence of E_{110}_, E_{111}_, E_{112}_ and E_{100}_; thus, the deformed grains with low energy storage cannot be the main nucleation sites, and they are always consumed by γ grains at the later recrystallization stage [14]. 

However, the nucleation of {001} 〈130〉 recrystallized grains in the {100} and {113} deformed matrix interior was observed in the 0.012Y sample. The sub-structured grains around the inclusions at the region of the high dislocation density can grow without the energy from the deformed matrix. Once sub-structured grains are formed around the large inclusions, they can grow by grain boundary migration and form a crystal nucleus [15]. It has been claimed that the preferred nucleation of grain has a particular crystallographic orientation relationship with the deformed matrix. 

At present, a large number of studies show that the {001} grain originates from λ oriented grains [16,17]. The large-size RE inclusions in {001} deformed grains with low energy storage are conducive to the recrystallization of favorable oriented grains. 

The driving force of recrystallization is mainly related to the disappearance of dislocations in the sub-boundary, and the inhomogeneous deformation zone is more likely to appear around the large-size particles, which can effectively promote recrystallization [18]. In order to quantitatively further explain the distribution of dislocation density around different inclusions during plastic deformation, the RD-ND (roll direction–normal direction) planes of 0 Y and 0.012 Y samples after 20% reduction were scanned by SEM and EBSD. 

**Figure 7:**
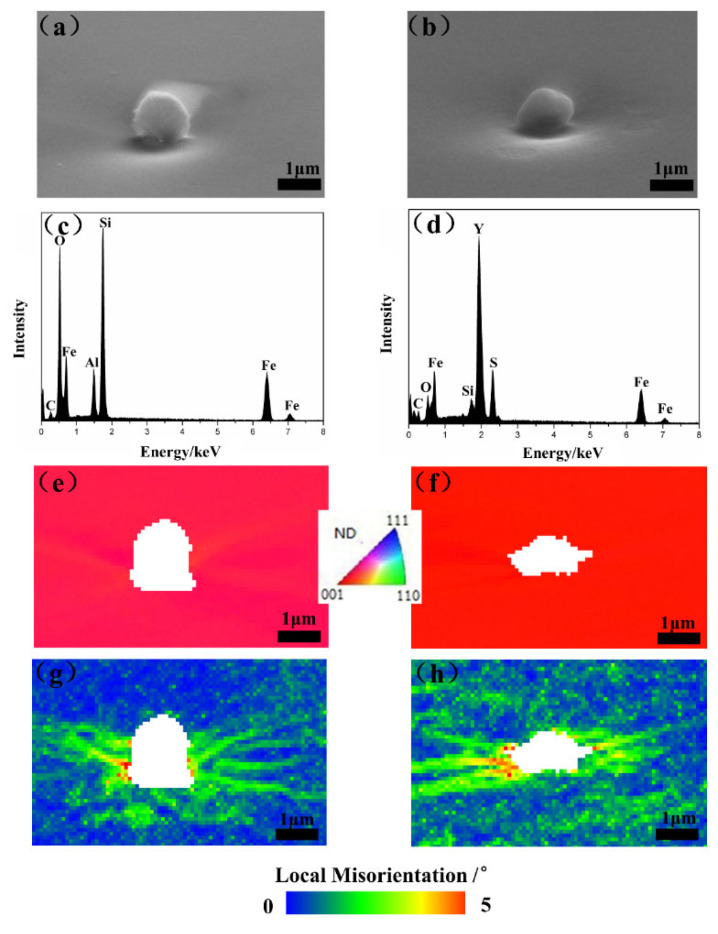
Micrographs around the Al_2_O_3_ (**a**,**c**,**e**,**g**) and Y_2_O_2_S (**b**,**d**,**f**,**h**) inclusion. (**a**,**b**) SEM micrograph; (**c**,**d**) EDS results; (**e**,**f**) IPF map; and (**g**,**h**) local misorientation map.

Figure 7a–d shows the micrographs and EDS results of different inclusions with the same size. IPF maps and the kernel average misorientation (KAM) are shown in Figure 7e–h. The stress concentration around Y_2_O_2_S was clearly higher than that of Al_2_O_3_ in the same {100} matrix. KAM can qualitatively reflect the degree of homogenization of plastic deformation. The relationship between geometrically necessary dislocation density (GND) and KAM is as follows [19]:

(1) $$\rho^{GND}=\frac{2KAM_{ave}}{\mu b}$$

where *μ* is the EBSD step size (0.1 μm), *b* is the Burgers vector (0.248 nm) and *KAM*_ave_ is the average local misorientation. All KAM values used for dislocation density calculation exclude KAM values greater than 3°, which are caused by grain boundaries rather than dislocation accumulation. By calculating the GND of the identical areas around the inclusion, we found that the dislocation density of 0Y sample was $3.215\times 10^{4}\;{\mathrm{cm}}^{-2}$, which was less than the dislocation density of $8.596\times 10^{4}\;{\mathrm{cm}}^{-2}$ of the 0.012Y sample. The dislocation density around the Y inclusion was much higher than for Al_2_O_3_. 

**Figure 8:**
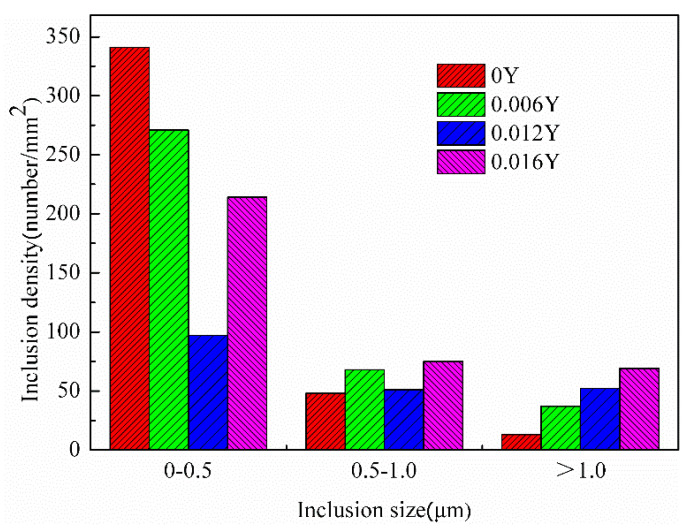
The inclusion size distribution of final sheets with different Y contents.

The inclusion size distribution of the final sheets is shown in Figure 8. The addition of Y reduced the number of fine inclusions, which promoted the grain growth. With the increase of the Y content, the number of inclusions larger than 1 μm continued to increase, which generated orientation gradients that provided sites for PSN. The addition of rare earth Y significantly coarsened and increased the number of large inclusions in the final sheets. RE can adsorb other harmful elements to form composite inclusions, and the hardness of RE oxysulfides is higher than that of Al_2_O_3_ and MnS [20]. 

Therefore, it is more likely to see rapid sub-boundary migration around coarse RE inclusions and the accumulation of more misorientation during the rolling process as well as high angle grain boundary (HAGB) formation during recrystallization [21]. Similarly, {114} 〈481〉 had widespread nucleation sites and was often found at the grain boundaries of α-fiber deformed grains [22], and the coarse Y inclusions provided additional nucleation sites for {114} 〈481〉. The increasing number of {114} 〈481〉 nucleation at the early stage of recrystallization impelled {114} 〈481〉, thereby, becoming one of the main textures in the 0.012 Y sample.

### 3.4. Nucleation and Growth of {001} 〈130〉 and {114} 〈481〉 Grains

**Figure 9:**
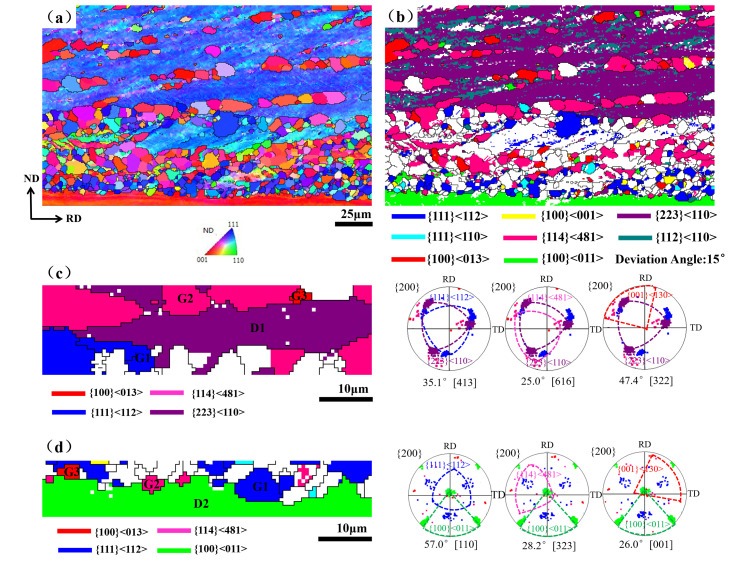
EBSD orientation maps in the partially recrystallized 0.012 Y sample. (**a**) IPF map; (**b**) texture component figure; (**c**) map of relationship between G1, G2 and G3 recrystallized grains and D1 deformed grains; and (**d**) map of relationship between G1, G2 and G3 recrystallized grains and D2 deformed grains.

Figure 9 shows the partial recrystallization maps of the 0.012 Y sample and the misorientation relationship between recrystallized grains and adjacent deformed grains. A large number of {114} 〈481〉 grains and a small amount of {001} 〈130〉 grains nucleated in the interior of {223} 〈110〉 deformed grains, and almost no {111} 〈112〉 grains appeared in {223} 〈110〉 deformed grains. Considering the strong {001} 〈130〉 and {114} 〈481〉 recrystallization textures obtained in the 0.012 Y sample, it is necessary to investigate the misorientation relationship between the newly nucleated grains and the deformed matrix. 

G1, G2 and G3 represented {111} 〈112〉, {114} 〈481〉 and {001} 〈130〉 recrystallized grains, respectively. D1 and D2 represented {223} 〈110〉 and {100} 〈011〉 deformed grains, respectively. We found that the misorientation relationships of G1, G2, G3 and D1 were 35.1° 〈413〉, 25.0° 〈616〉 and 47.4° 〈322〉, respectively. The misorientation relationship of {114} 〈481〉 recrystallized grains was close to the high mobility of Σ 19 grain boundary [23]. The {114} 〈481〉 grains had the advantage of nucleation site and quantity in the interior of the {223} 〈110〉 deformed grains, which were easier to grow. 

The misorientation relationships of G1, G2, G3 and D2 were 57.0° 〈110〉, 28.2° 〈323〉 and 26.0° 〈001〉, respectively. Since the 20–45° grain boundaries in BCC metal have high mobilities [24,25], {001} 〈130〉 and {114}〈481〉 recrystallized grains can grow rapidly in the {100} 〈011〉 deformed matrix. The oriented growth became the main mechanism for the formation of {001} 〈130〉 and {114} 〈481〉 recrystallization textures in the 0.012 Y sample. 

## 4. Conclusions

The addition of Y decreased the {111} 〈112〉 and enhanced the {001} 〈130〉 and {114} 〈481〉 texture components in annealed 4.5 wt% Si steel sheets. The segregation of Y restrained the advantage of {111} nucleation at grain boundaries, which was beneficial to the nucleation of other oriented grains elsewhere. Compared with Al_2_O_3_ inclusion, Y_2_O_2_S inclusion with the same size produced more distortion in the matrix under the same strain and accumulated larger misorientation, making the deformed grains with low energy storage nucleate faster at early recrystallization stage, which provided more preferential nucleation sites of {223} 〈110〉 and {001} deformed grains for {001} 〈130〉 and {114} 〈481〉 grains. Strong {001} 〈130〉 and {114} 〈481〉 recrystallization textures formed in the samples containing 0.012 wt% Y due to more nucleation sites and the high mobility of grain boundaries.

## Figures and Tables

**Table 1:** The chemical composition of the ingots (wt%).

Sample	Y	Si	C	S	O	N	Fe
0 Y	0	4.5	0.0025	0.0023	0.020	0.0014	Bal.
0.006 Y	0.006	4.5	0.0029	0.0024	0.0017	0.0017	Bal.
0.012 Y	0.012	4.5	0.0028	0.0018	0.0017	0.0017	Bal.
0.016 Y	0.016	4.5	0.0025	0.0019	0.0016	0.0016	Bal.

## Data Availability

Not applicable.

## References

[1] Zhang B., Liang Y.F., Wen S.B., Wang S., Shi X.J., Ye F., Lin J.P. (2019). High-strength low-iron-loss silicon steels fabricated by cold rolling. J. Magn. Magn. Mater..

[2] Zu G.Q., Zhang X.M., Zhao J.W., Wang Y.Q., Yan Y., Li C.G., Cao G.M., Jiang Z.Y. (2017). Fabrication and properties of strip casting 4.5 wt% Si steel thin sheet. J. Magn. Magn. Mater..

[3] Li F., Li H., Wu Y., Zhao D., Peng B., Huang H., Zheng S., You J. (2017). Effect of precipitates on grain growth in non-oriented silicon steel. J. Mater. Res..

[4] Hou C.K., Liao C.C. (2008). Effect of Cerium Content on the Magnetic Properties of Non-oriented Electrical Steels. ISIJ Int..

[5] Wan Y., Chen W.Q., Wu S.J. (2014). Effects of Lanthanum and Boron on the Microstructure and Magnetic Properties of Non-oriented Electrical Steels. High Temp. Mater. Proc..

[6] Wan Y., Chen W.Q., Wu S.J. (2013). Effect of lanthanum content on microstructure and magnetic properties of non-oriented electrical steels. J. Rare Earth..

[7] Shi C.J., Jin Z.L., Ren H.P., You J.L. (2017). Effect of lanthanum on recrystallization behavior of non-oriented silicon steel. J. Rare Earth..

[8] Qin J., Zhou Q.Y., Zhao H.B., Zhao H.J. (2021). Effects of yttrium on the microstructure, texture, and magnetic properties of non-oriented 6.5 wt% Si steel sheets by a rolling process. Mater. Res. Express.

[9] He Z.H., Sha Y.H., Gao Y.K., Chang S.T., Zhang F., Zuo L. (2020). Recrystallization texture development in rare-earth (RE)-doped non-oriented silicon steel. J. Iron Steel Res. Int..

[10] Park J.T., Szpunar J.A. (2003). Evolution of recrystallization texture in nonoriented electrical steels. Acta Mater..

[11] Barnett M.R., Kestens L. (1999). Formation of {111} <110> and {111} <112> Textures in Cold Rolled and Annealed IF Sheet Steel. ISIJ Int..

[12] Wang L.M., Qin L., Ji J.W., Lan D.N. (2006). New study concerning development of application of rare earth metals in steels. J. Alloy. Compd..

[13] Li L.F., Yang W.Y., Sun Z.Q. (2013). Dynamic Recrystallization of Ferrite with Particle-Stimulated Nucleation in a Low-Carbon Steel. Met. Mater. Trans. A.

[14] Zu G.Q., Zhang X.M., Zhao J.W., Wang Y.Q., Cui Y., Yan Y., Jiang Z.Y. (2016). Analysis of {411}<148>recrystallisation texture in twin-roll strip casting of 4.5 wt% Si non-oriented electrical steel. Mater. Lett..

[15] Wang X.Y., Jiang J.T., Li G.A., Wang X.M., Shao W.Z., Zhen L. (2021). Particle-stimulated nucleation and recrystallization texture initiated by coarsened Al2CuLi phase in Al–Cu–Li alloy. J. Mater. Res. Technol..

[16] Xu Y.B., Zhang Y.X., Wang Y., Li C.G., Cao G.M., Liu Z.Y., Wang G.D. (2014). Evolution of cube texture in strip-cast non-oriented silicon steels. Scr. Mater..

[17] Cheng L., Zhang N., Yang P., Mao W.M. (2012). Retaining {100} texture from initial columnar grains in electrical steels. Scr. Mater..

[18] Rios P.R., Jr F.S., Sandim H.R.Z., Plaut R.L., Padilha A.F. (2005). Nucleation and growth during recrystallization. Mat. Res..

[19] Liu D.F., Qin J., Zhang Y.H., Wang Z.G., Nie J.C. (2020). Effect of yttrium addition on the hot deformation behavior of Fe–6.5 wt%Si alloy. Mat. Sci. Eng. A.

[20] Yang C.Y., Luan Y.K., Li D.Z., Li Y.Y. (2019). Effects of rare earth elements on inclusions and impact toughness of high-carbon chromium bearing steel. J. Mater. Sci. Technol..

[21] Robson J.D., Henry D.T., Davis B. (2009). Particle effects on recrystallization in magnesium–manganese alloys: Particle-stimulated nucleation. Acta Mater..

[22] Li H.Z., Liu Z.Y., Wang X.L., Ren H.M., Li C.G., Cao G.M., Wang G.D. (2017). {114} <481> Annealing texture in twin-roll casting non-oriented 6.5 wt% Si electrical steel. J. Mater. Sci..

[23] Sanjari M., He Y.L., Hilinski E.J., Yue S., Kestens L.A.I. (2016). Development of the {113}<uvw>texture during the annealing of a skew cold rolled non-oriented electrical steel. Scr. Mater..

[24] Rajmohan N., Szpunar J.A. (2001). An analytical method for characterizing grain boundaries around growing goss grains during secondary recrystallization. Scr. Mater..

[25] Zhang Y.H., Yang J.F., Qin J., Zhao H.B. (2021). The effect of grain size before cold rolling on the magnetic properties of thin-gauge non-oriented electrical steel. Mater. Res. Express.

